# Coactosin-Like 1 Antagonizes Cofilin to Promote Lamellipodial Protrusion at the Immune Synapse

**DOI:** 10.1371/journal.pone.0085090

**Published:** 2014-01-13

**Authors:** Joanna Kim, Michael J. Shapiro, Adebowale O. Bamidele, Pinar Gurel, Puspa Thapa, Henry N. Higgs, Karen E. Hedin, Virginia S. Shapiro, Daniel D. Billadeau

**Affiliations:** 1 Department of Biochemistry and Molecular Biology, College of Medicine, Mayo Clinic, Rochester, Minnesota, United States of America; 2 Department of Immunology, College of Medicine, Mayo Clinic, Rochester, Minnesota, United States of America; 3 Department of Molecular Pharmacology and Experimental Therapeutics, College of Medicine, Mayo Clinic, Rochester, Minnesota, United States of America; 4 Department of Biochemistry, Geisel School of Medicine at Dartmouth, Dartmouth College, Hanover, New Hampshire, United States of America; Helmholtz Centre for Infection Research, Germany

## Abstract

Actin depolymerizing factor-homology (ADF-H) family proteins regulate actin filament dynamics at multiple cellular locations. Herein, we have investigated the function of the ADF-H family member coactosin-like 1 (COTL1) in the regulation of actin dynamics at the T cell immune synapse (IS). We initially identified COTL1 in a genetic screen to identify novel regulators of T cell activation, and subsequently found that it associates with F-actin and localizes at the IS in response to TCR+CD28 stimulation. Live cell microscopy showed that depletion of COTL1 protein impaired T cell spreading in response to TCR ligation and abrogated lamellipodial protrusion at the T cell – B cell contact site, producing only a band of F-actin. Significantly, re-expression of wild type COTL1, but not a mutant deficient in F-actin binding could rescue these defects. In addition, COTL1 depletion reduced T cell migration. *In vitro* studies showed that COTL1 and cofilin compete with each other for binding to F-actin, and COTL1 protects F-actin from cofilin-mediated depolymerization. While depletion of cofilin enhanced F-actin assembly and lamellipodial protrusion at the IS, concurrent depletion of both COTL1 and cofilin restored lamellipodia formation. Taken together, our results suggest that COTL1 regulates lamellipodia dynamics in part by protecting F-actin from cofilin-mediated disassembly.

## Introduction

The actin cytoskeleton participates in many cellular processes including immune synapse (IS) formation during T cell activation [1]. Upon interaction of the T cell antigen receptor (TCR) with peptide-major histocompatibility complexes on the surface of antigen presenting cells (APCs), round T cells produce a lamellipodial protrusion at the IS that is reminiscent of migrating cells and is highly dependent upon actin cytoskeleton rearrangement [2], [3], [4]. We have previously demonstrated that membrane protrusion and filamentous (F)-actin accumulation at the T cell–APC contact site requires Arp2/3-dependent branched F-actin generation [5], as well as the Arp2/3 nucleation-promoting factor, WAVE2 [6]. In addition, WASP, mDia1, IQGAP1, HS1 and several other proteins have been shown to participate in F-actin remodeling and stabilization at the IS [5], [7], [8]. Since it is generally appreciated that F-actin reorganization is essential for proper APC recognition, IS formation and efficient signaling leading to T cell activation, it is important to understand and identify key regulators of this highly dynamic process and their impact on T cell function.

The generation of lamellipodia for directed cell migration is a highly coordinated process. The dendritic nucleation treadmilling model proposes several steps whereby actin filament formation and turnover are coupled in order to generate and sustain the growing lamellipodial structure [9], [10]. This includes rapid elongation of barbed ends through the addition of profilin-ATP-actin [11], which pushes the membrane forward and termination of F-actin growth through the binding of F-actin capping proteins [12]. In addition, cofilin, an actin depolymerizing factor-homology (ADF-H) family member severs ADP-F-actin via conformational changes in filament structure and depolymerizes aged filaments at the pointed ends [13]. Together, this dynamic process of filament nucleation, severing and depolymerization synergize to produce a large pool of new actin barbed ends and free actin monomers at the leading edge that support and maintain lamellipodial protrusion. Based on this information, it might be expected that the actin severing and depolymerizing activity of cofilin would be required to promote or maintain lamellipodia formation, but in fact, depletion of cofilin results in expanded lamellipodial protrusion in several cell models [14], [15], [16] suggesting that in some cellular systems cofilin regulates actin filament dynamics by accelerating actin filament disassembly.

There are three distinct groups of ADF-H family members which include ADF/cofilin, twinfilins and Abp1/drebrins [17]. While the cellular roles of cofilin have been well studied, the functions of the other family members in regulating F-actin dynamics in T cells have not. Coactosin like protein 1 (COTL1), is a member of the ADF/cofilin family and is highly related to the actin-binding protein coactosin which was first identified in *Dictyostelium discoideum* [18]. Despite having low amino acid sequence identity with cofilin, COTL1 and cofilin have highly homologous structures, which impart their binding propensities for F-actin or globular (G)-actin through their respective ADF-H domains [19]. COTL1 was shown to bind F-actin, but not G-actin and site-directed mutagenesis has determined that lysine 75, located in the ADF-H domain plays a critical role in binding to F-actin [20], [21], [22], [23], [24]. While biochemical and structural studies have been performed on COTL1, the cellular functions as they pertain to the regulation of F-actin dynamics have not been elucidated. In this paper, we identify COTL1 as a potential regulator of T cell activation. Moreover, we show that COTL1 is recruited to the IS where it is required for lamellipodial protrusion. Mechanistically, we show that COTL1 and cofilin compete for binding to F-actin and that COTL1 attenuates cofilin-mediated F-actin depolymerization. Our data suggest a model whereby COTL1 binding to F-actin functions to limit cofilin-mediated F-actin severing during lamellipodia generation.

## Materials and Methods

### Reagents, Antibodies and Plasmids

An antibody against COTL1 was obtained by immunization of rabbits with GST-COTL1 protein (Cocalico Biologicals). Rabbit anti-cofilin was purchased from Cytosekeleton Inc. Anti-β actin was from Sigma-Aldrich. Anti-Zap70 has been described previously [25], [26]. Anti-human CD3 (OKT3) was purchased from the Mayo Pharmacy and anti-human CD28 was purchased from BD Biosciences. Staphylococcal Enterotoxin A, B, C, and E were from Toxin technology Inc. The shRNA suppression vector, pFRT.H1p and the suppression/re-expression vectors, pCMS3.mCherry.H1p and pCMS3.eGFP.H1p have been described previously [5], [27]. The siCOTL1 targeting sequence is 5′-GGGATTGTAAAGAACATCT-3′ corresponding to nucleotides 1758−1776 in the 3′ UTR using National Center for Biotechnology Information Genbank accession number NM_021149 (http://www.ncbi.nlm.nih.gov/genbank/). COTL1 was amplified from a cDNA library and was mutated at R73E, K75E to generate a COTL1 protein deficient in F-actin binding (referred to as a non-actin-binding mutant, ABM) [24].

### Retroviral library transduction and screening

A human leukocyte cDNA retroviral library was purchased from BD Biosciences (Cat# HL8007BB) and retroviruses were generated according to the manufacturer’s instructions. This retroviral library was used to transduce the J.REM 474 Jurkat mutant cell line and screened for expression of GFP, as was previously described [28]. The COTL1 retroviral insert was identified by amplification of genomic DNA from rescued clones with pLIB primers (BD Clontech).

### Luciferase Reporter Assays

Transfections, stimulations and luciferase assays were performed as previously described [29]. In brief, 1.5×10^7^ Jurkat T cells were washed once and resuspended in 0.4 ml of serum-free RPMI 1640 media. 20 µg of RE/AP luciferase reporter was used per transfection. Electroporation was performed using a Gene Pulser II (Bio-Rad) at 250 V, 950 µF. Cells were resuspended in 10 ml of RPMI 1640 media supplemented with 5% FCS (Invitrogen). The following day, live cells were counted by trypan blue exclusion (Bio-Whittaker), and 1×10^5^ cells per sample were stimulated as denoted in the figures. Cells were left unstimulated or stimulated with anti-TCR (C305, 1/1000 final dilution) and anti-CD28 (1 µg/ml) for 7 h. Luciferase assays were performed as previously described [29].

### CD4^+^ T cell isolation, cell culture and transfection

Primary human peripheral blood (Mayo Clinic Blood Donor Center) CD4^+^ T cells were isolated with RosetteSep Human CD4^+^ T cell Enrichment cocktail (Stemcell Technologies) as described by manufacturer. T cell blasts were generated by the addition of 5 µg/ml phytohemagglutinin (day 1) and cultured in 20 U/ml proleukin (Chiron corporation) for additional 5 days. Human primary CD4^+^ T cells, Jurkat T cells (T1B-152, American type culture collection (ATCC)), Jurkat T cells stably expressing GFP-actin [5] and Raji B cells (CCL-86, ATCC) were cultured in RPMI 1640 media supplemented with 5% FBS, 5% BCS and 1% L-glutamine. For transient suppression and suppression/re-expression, 1×10^7^ Jurkat T cells were transfected with 40 µg of suppression or suppression/re-expression plasmids using a BTX ECM 830 electroporator (315 V, 10 ms, 1 pulse). Transfected Jurkat T cells were allowed to proliferate for 3 d prior to being used in experiments.

### Immunoblotting

Cell lysates (70-100 µg) were resolved on Tris-glycine SDS-PAGE gels, transferred to PVDF membranes and blocked in 4% BSA in TBS. Target proteins were probed with primary antibodies in 2% BSA in TBST. Bound antibodies were detected with goat anti-mouse IgG (Santa Cruz technology) or goat anti-rabbit IgG (Cell Signaling Technology) conjugated with horseradish peroxidase and SuperSignal (Pierce).

### F-actin enriched fraction assay

Jurkat T cells (5×10^6^) were suspended in serum-free RPMI 1640 media containing anti-CD3 antibody (OKT3) and anti-CD28 antibody (5 µg/ml each) and incubated for 10 min on ice. After centrifugation, cells were resuspended in serum-free media with 20 µg/ml goat anti-mouse IgG (MP Biomedicals, LLC) for cross-linking and incubated at 37°C for various time points. The F-actin enriched pellet was isolated as previously described [30]. Supernatant and F-actin rich fractions were separated on Tris-glycine SDS-PAGE gels and COTL1 and cofilin were detected by immunoblotting.

### Protein preparation and purification

Recombinant GST-COTL1 WT, GST-COTL1 ABM and GST-cofilin were expressed in bacteria, and purified as previously described [31]. GST was removed using the Thrombin CleanCleave kit as per the manufacturers instructions (Sigma-Aldrich).

### Migration assay

Jurkat T cells were transfected with pCMS3.H1p vector or pCMS3.H1p.shCOTL1 plasmids. 72 h later, migration assays were performed using the YFP fluorescence expressed by these plasmids to detect migrating cells. All migration assays were performed in 96-well Chemotax chemotaxis plates (Neuroprobe, Gaithersburg, MD) with 5 µm pore filters coated with fibronectin (Invitrogen). 5×10^5^ Jurkat T cells were diluted in migration buffer (RPMI 1640 media without phenol red supplemented with 0.5% BSA and 1% DMSO) and placed in the upper chamber of each well. After migrating toward the lower chamber containing the indicated concentrations of SDF-1 for 1 h at 37°C, cells remaining in the upper chambers were removed and cells that migrated into the lower chambers were quantified by measuring YFP (485 nm excitation/530 nm emission) fluorescence using a Cytofluor 4000 spectrometer (PerSeptive Biosystems, Framingham, MA).

### Actin polymerization/depolymerization by fluorescence spectroscopy

Actin and pyrene-labeled actin were prepared as described [32]. For actin polymerization, unlabeled and pyrene-labeled actin were mixed (1.1 µM, 5% pyrene-labeled actin) in G-buffer (2 mM Tris-HCl, pH 8.0, 0.5 mM DTT, 0.2 mM ATP, 0.1 mM CaCl_2_, 10 mM MgCl_2_, and 0.01% NaN_3_) to generate the pyrene-actin stock. This stock was changed to Mg^2+^ salt by incubation in 1 mM EGTA and 0.1 mM MgCl_2_ for 2 min at 23°C immediately before polymerization. Actin was polymerized by the addition of 10× KMEI buffer to a concentration of 1× (50 mM KCl, 1 mM MgCl_2_, 1 mM EGTA and 10 mM imidazole pH 7.0), with the remaining volume made up by G-Mg. For F-actin depolymerization, 1.1 µM actin (5% pyrene-actin) was polymerized for 2 h at 23°C and 45 µl of pyrene-F-actin was mixed with 5 µl of different combinations of Latrunculin B (Lat B), cofilin and/or COTL1. Pyrene F-actin fluorescence was monitored at 365 nm excitation and 410 nm emission in a 96-well fluorescence plate reader (Tecan Infinite M1000, Mannedorf, Switzerland) every 3 s. These data were subsequently analyzed using Kaleidagraph (Synergy Software, Reading, PA) and expressed as pyrene F-actin fluorescence. The time delay between mixing of final components and collecting the first fluorimeter data readings ranged between 15 and 20 s. Each experiment was performed with triplicates and three independent experiments were performed [33].

### Immunofluorescence microscopy

Conjugates were formed for 10 min and plated onto poly-L-lysine coated coverslips for 5 min at 37°C. For immunofluorescence staining, slides were fixed with 4% paraformaldehyde for 10 min, permeablized with 0.15% Triton X-100 and incubated in blocking buffer for 30 min, followed by incubation with primary antibodies overnight at 4°C. Coverslips were washed 4 times in PBS and then incubated with FITC-conjugated secondary antibodies with or without rhodamine-phalloidin or AlexaFluor 647-phalloidin (for F-actin) for 1 h at room temperature. Coverslips were washed extensively in PBS and then mounted using SlowFade Gold anti-fade reagent (Invitrogen). Images were captured using a Zeiss LSM 710 confocal or Zeiss Axiovert 200 M microscope. For quantification of F-actin accumulation at the IS, conjugates of GFP^+^ Jurkat T cells and CMAC-stained Raji B cells were randomly selected and scored for the presence of accumulated F-actin at the cell-cell contact site as previously described [7]. At least 100 conjugates were counted in each of three independent experiments.

### Live cell imaging

For F-actin spreading analysis, 4-well Lab-Tek II chambered cover glasses (Nunc) were coated with 400 µl of OKT3 (25 µg/ml in PBS) by gently rocking overnight at 4°C and washed with PBS. 400 µl of pre-warmed (37°C) RPMI 1640 medium was subsequently added to the wells. The CO_2_ Module on the live cell imagining system maintained a 5% CO_2_ environment and the Heating Insert P Lab-Tek Compact objective heater on the microscope stage, which was controlled by the TempModule and Heating Unit XLS maintained a constant temperature of 37°C. The Zeiss Definite Focus System was used to capture time-lapse cell images. Jurkat T cells stably expressing GFP-actin were transiently transfected with pCMS3.mCherry.H1p vector and cultured for 60–72 h. Cells were harvested and resuspended at 2–3×10^6^ cells/ml in RPMI 1640 media. 5 µl of cells were dropped above the bottom of the wells and time-lapse series of randomly selected cherry positive cells were acquired on an inverted Zeiss LSM 710 with a C Apo 63X/1.2W DICIII objective. Stacks of 2 images spaced at 0.3 mm, starting from the plane of the coverglass, were collected with a new stack initiated every 20 s for a total of 33 min. Video series were aligned on the basis of initial contact with the coverslip and the average of the cell area (pixels) as measured at each time point using Image J (rsbweb.nih.gov/ij/). For lamellipodial protrusion at the cell-cell contact site, 35 mm glass bottom microwell dishes (MatTek cooperation) was coated with poly-L-lysine overnight at 4°C and washed with water for 1 h. Raji B cells stained with CMAC and loaded with SEE were harvested and resuspended at 1×10^6^ cells/ml. Six hundred microliter of cells were spread on the glass bottom, incubated for 10 min, and then 2 ml of RPMI 1640 media were replaced. Transfected Jurkat T cells prepared as described above were added and imaging was initiated when Jurkat T cell made contact with Raji B cell. Time-lapse images were collected for a total of 33 min. Time-compressed movies were generated in Quicktime with an average frame-speed of 4 frames per second.

## Results

### Identification of COTL1 as a potential novel regulator of T cell activation using a genetic complementation screen

**Figure 1:**
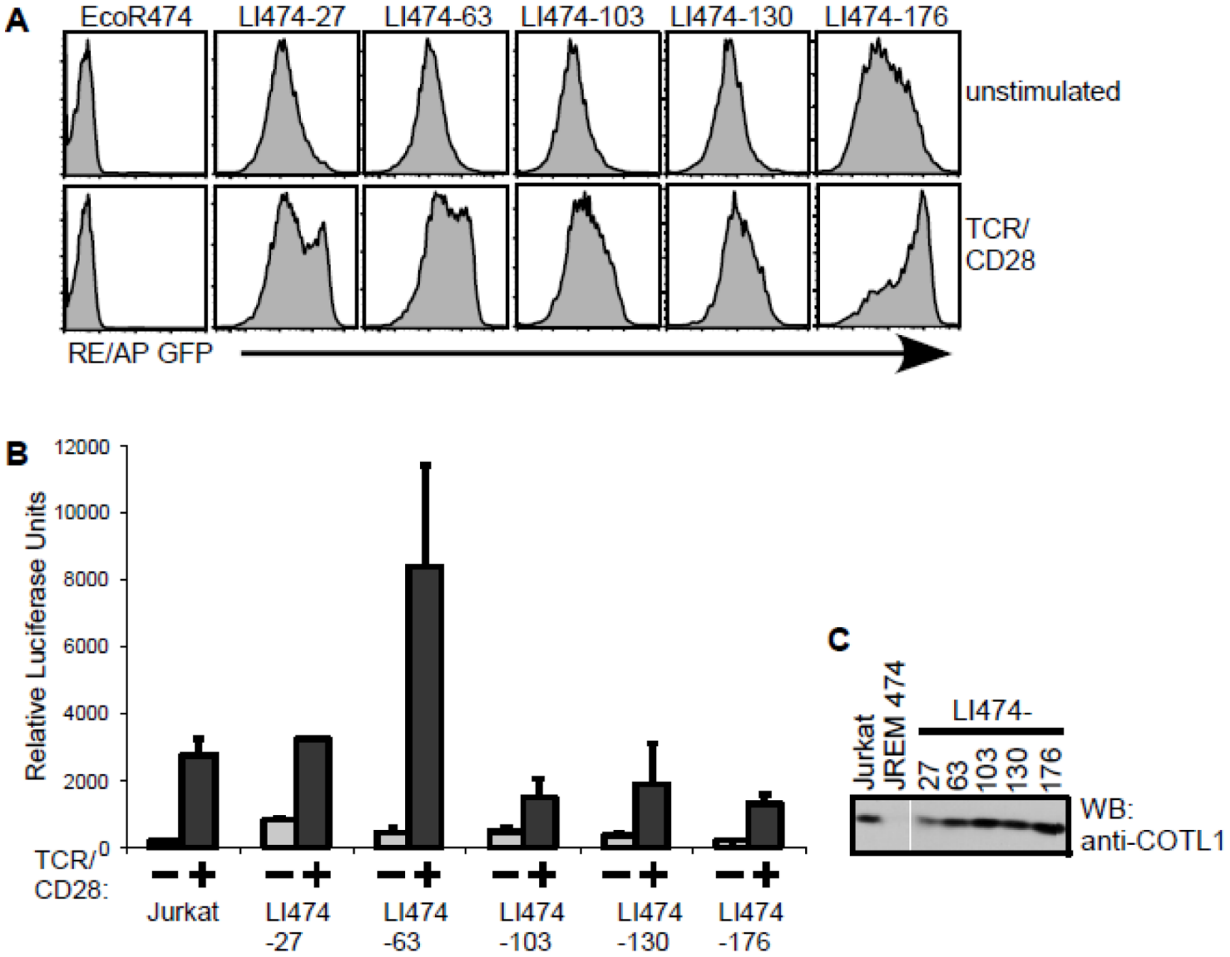
COTL1 was identified as a regulator of T cell activation by a genetic complementation screen. (**A**) Shown is the screening for restoration of RE/AP GFP reporter expression in a T cell activation defective Jurkat T mutant cell line (J.REM 474) expressing the Ecotropic receptor for retroviral transduction (EcoR 474) and 5 retrovirally transduced clones in which GFP expression is restored (LI474-27, LI474-63, LI474-103, LI474-130, LI474-176). The mutant J.REM474 cell line does not express the reporter with or without TCR+CD28 stimulation. The five rescued clones all constitutively express GFP and demonstrate upregulation with TCR+CD28 stimulation. (**B**) Confirmation of restored activation in the transduced clones. Jurkat T cells and each of the clones identified above were transfected with 20 µg RE/AP luciferase reporter, and examined for activation by TCR+CD28. The results shown are the average of two independent transfections, and error bars reflect standard deviation of the mean. (**C**) Immunoblot showing expression of COTL1 in the wild-type Jurkat T cell line, J.REM 474 and in rescued clones LI474-27, LI474-63, LI474-103, LI474-130, LI474-176.

Previously, we utilized a genetic complementation screen to identify novel regulators of T cell activation, and identified the transcriptional repressor NFκB activating protein (NKAP), which is critical for T cell development and maturation [28], [34], [35]. As the first gene identified by this screen, NKAP provided a proof-of-principle for the approach, we repeated the screen to identify additional molecules. A Jurkat mutant cell line in which T cell activation was defective which contained an integrated RE/AP GFP reporter [36] was transduced with a retroviral leukocyte library and screened for clones in which activation of the GFP reporter was restored. Five of the clones in which reporter activation was restored (Figure 1A and B) were found to retrovirally express the same full length cDNA for COTL1 (coactosin-like 1). We examined the expression of COTL1 by immunoblotting in the original Jurkat T cell line, the JREM 474 Jurkat mutant cell line, and the five clones which contained a COTL1-expressing retrovirus (Figure 1C). COTL1 is expressed in wild type Jurkat T cells, but is decreased in the JREM 474 Jurkat mutant cell line. The five cell lines in which signaling was restored re-express COTL1 at levels equivalent to wild type Jurkat T cells. Therefore, loss of COTL1 expression may be the cause of the T cell activation defect in JREM 474, implying that COTL1 is required for T cell activation.

### COTL1 and F-actin are co-localized to the immune synapse in response to TCR+CD28 stimulation

**Figure 2:**
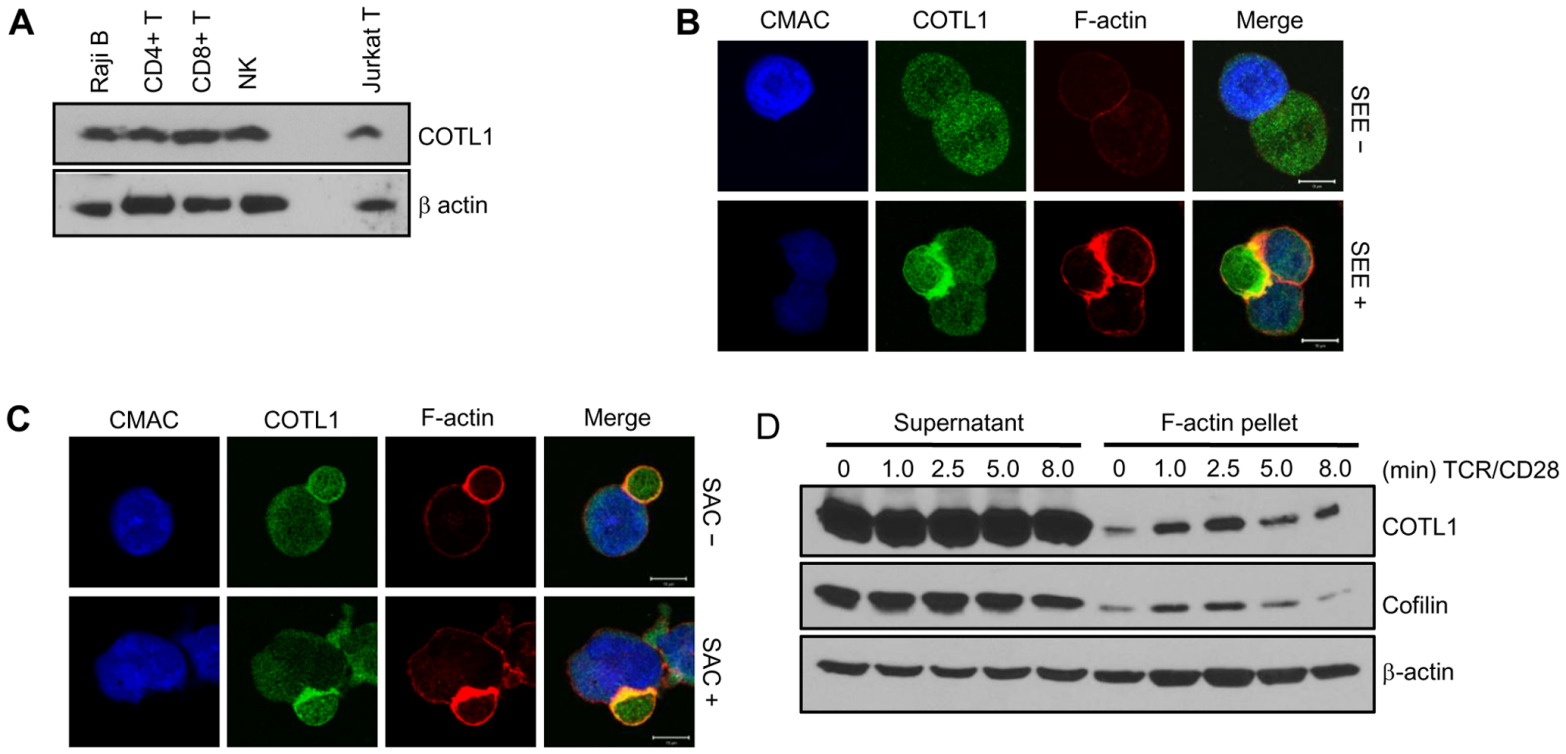
COTL1 and F-actin are accumulated to Immune Synapse (IS) in response to TCR+CD28 stimulation. (**A**) Cell lysates were prepared from the indicated cell lines, separated on Tris-glycine SDS-PAGE gels and immunoblotted for COTL1 and β-actin. Jurkat (**B**) or human CD4+ T cells (**C**) were incubated with CMAC-stained Raji B cells unloaded or loaded with SEE or SAC. Cell conjugates were subsequently fixed, stained for COTL1 and F-actin and visualized by confocal microscopy. (**D**) Jurkat T cells were costimulated with anti-CD3+CD28 antibodies for the indicated time points and supernatant and the F-actin rich pellet were isolated. Immunoblot analysis against COTL1, cofilin and β-actin were performed. Scale bar; 10 µm.

Coactosin, the first ortholog described, was isolated from *Dictyostelium* as an F-actin binding protein [18] and COTL1 was identified as a human F-actin binding protein with significant homology to coactosin [20]. Thus, we decided to examine the role of COTL1 in F-actin dynamics in T cells. As shown in Figure 2A, COTL1 is highly expressed in various lymphoid cell lines. Since COTL1 has been shown to bind F-actin, we next determined whether COTL1 localizes with F-actin at the IS. In fact, we found that COTL1 accumulated at the cell-cell contact site formed between Jurkat T cells and Raji B cells pulsed with staphylococcal enterotoxin E (SEE) (Figure 2B) as well as CD4^+^ T cells and Raji B cells pulsed with superantigen cocktail (SAC) (Figure 2C). Significantly, preparation of F-actin enriched fractions from Jurkat T cells following TCR+CD28 ligation showed a stimulation-dependent accumulation of F-actin, COTL1 and cofilin that began as soon as 1 min post stimulation and persisted for up to five min (Figure 2D). Taken together, these data indicate that COTL1 F-actin binding can be induced by TCR+CD28 triggering and that COTL1 localizes with polymerized F-actin at the IS.

### COTL1 is required for F-actin protrusion at the immune synapse

**Figure 3:**
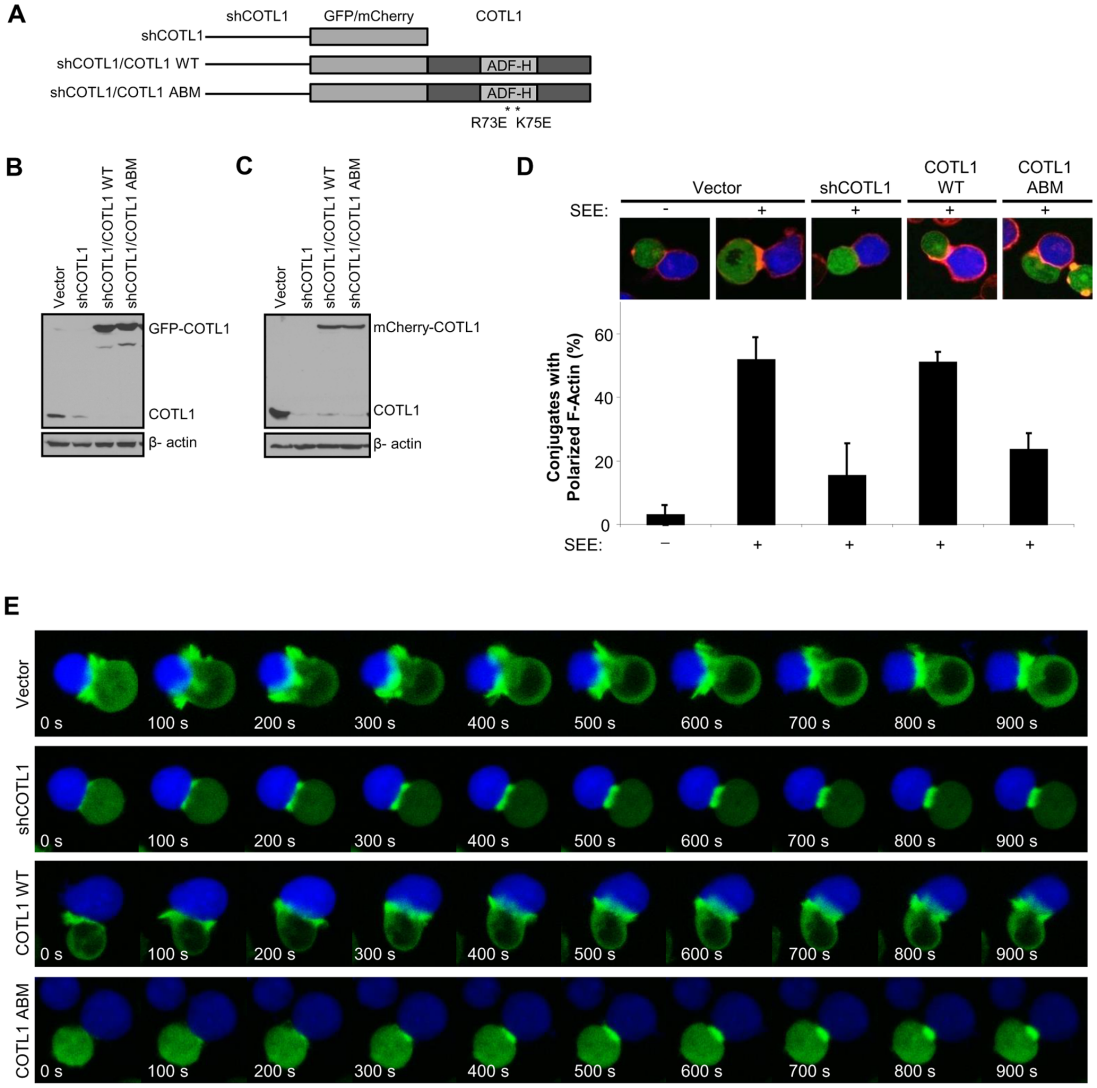
COTL1 is required for lamellipodial protrusion at the immune synapse. (**A**) Cartoon depicting the suppression and suppression/re-expression plasmids utilized in these studies. (**B, C**) Immunoblot analysis showing that endogenous COTL1 protein was suppressed and fluorescent-tagged COTL1 WT and COTL1 ABM were reconstituted, respectively. (**D**) Jurkat T cells were transfected with the GFP-expressing suppression/re-expression plasmids shown in (**A**). 72 h post transfection they were conjugated with CMAC-stained Raji B cells loaded or unloaded with SEE. Cell conjugates were stained for F-actin and imaged using fluorescence microscopy. Representative images were shown (**D**, top panels) and F-actin accumulation at the synapse was scored and quantification was displayed (**D**, lower graph). Over 100 conjugates were scored in 3 independent experiments. Error bars represent SEM. (**E**) Jurkat T cells stably expressing GFP-actin were transfected with mCherry suppression/re-expression plasmids shown in (**A**) and cultured for 3 d. For live cell imaging, RPMI 1640 media containing CMAC-stained SEE-loaded Raji B cells were added to Poly-L-lysine-coated coverglass chamber and incubated for 10 min. Afterwards, Jurkat T cells were dropped above the chamber and cherry positive Jurkat T cells were monitored by confocal microscopy every 20 s for 30 min following Jurkat T-Raji B cell contact for GFP-actin enriched lamellipodial protrusion. Image sequences captured every 100 s were shown. See associated Movies S1-S4.

To further investigate whether COTL1 regulates actin cytoskeleton rearrangement at the IS, COTL1 was suppressed and wild type (WT) or an R73E/K75E mutant incapable of binding to F-actin (referred to as a non-actin-binding mutant, ABM) were reconstituted using the pCMS3.mCherry.H1p or pCMS3.eGFP.H1p suppression/re-expression vector system (Figure 3A). As expected, 3 d post transfection, COTL1 was efficiently depleted and eGFP and mCherry protein tagged-COTL1 proteins were successfully reconstituted (Figure 3B and 3C). We next examined whether the depletion of COTL1 would affect F-actin accumulation at the IS. To do this, Jurkat T cells were transfected with eGFP expressing suppression/re-expression plasmids and conjugated with CMAC-stained SEE-pulsed Raji B cells. Fixed conjugates were stained with rhodamine-phalloidin for F-actin and accumulation at the IS was scored. As expected, there was little F-actin accumulation at the IS in empty vector-transfected cells stimulated with unpulsed-Raji B cells (Figure 3D). However, F-actin accumulation was seen in nearly 60% of Jurkat T cell–SEE-pulsed Raji B cell conjugates. Significantly, depletion of COTL1 resulted in a substantial loss of polymerized F-actin at the IS, whereas re-expression of WT COTL1 rescued F-actin accumulation back to empty vector-transfected levels (Figure 3D). In contrast, re-expression of COTL1 ABM was unable to restore F-actin accumulation at the IS (Figure 3D), suggesting that COTL1 binding to F-actin is required for the accumulation of F-actin at the T cell–B cell contact site.

To examine the effect of COTL1 depletion on F-actin cytoskeletal dynamics in real time, we imaged mCherry-positive GFP-actin expressing Jurkat T cells in response to contact with SEE-pulsed Raji B cells. In Jurkat T cells transfected with empty vector, GFP-actin accumulated at the contact site shortly after the initial contact leading to the development of an F-actin rich lamellipodial protrusion over the interface for up to 10 min that gradually retracted forming an F-actin-rich band at the contact site (Figure 3E and Movie S1). In contrast, while Jurkat T cells depleted of COTL1 did accumulate a band of GFP-actin at the IS, they failed to generate defined lamellipodia during the entire imaging period (Figure 3E and Movie S2). Importantly, reconstitution with WT COTL1 restored lamellipodia formation at the IS, whereas reconstitution with COTL1 ABM showed a similar inability to generate F-actin-rich lamellipodia at the IS (Figure 3E and Movies S3 and S4). Taken together, these data suggest that COTL1 is required for lamellipodial protrusion at the IS that requires its ability to bind F-actin.

### COTL1 regulates TCR-stimulated F-actin spreading and chemokine-dependent cell migration

In Figure 3 we showed that COTL1-depleted Jurkat T cells have impaired lamellipodial protrusion toward the APC and a defect in F-actin accumulation at the IS. As this could be a result of diminished cell-cell adhesion, and we have previously shown that WAVE2-generated F-actin is required for efficient integrin-mediated adhesion [6], [37] we examined whether impaired actin cytoskeletal dynamics by COTL1 suppression reduced cellular adhesion. First, we examined whether Rap1 activation, which is required for integrin clustering and affinity maturation was affected by COTL1 depletion. Using GST-RalGDS to capture active Rap1-GTP, we found that Rap1-GTP levels increased similarly in both empty vector-transfected and COTL1-depleted Jurkat T cells following TCR+CD28 stimulation (Figure S1A). Consistent with this finding, Jurkat T cell-Raji B cell conjugate formation was not affected by loss of COTL1 (Figure S1B). Taken together, these data suggest that the inability to generate lamellipodial structure at the T cell-APC interface is not due to defective integrin-mediated adhesion.

**Figure 4:**
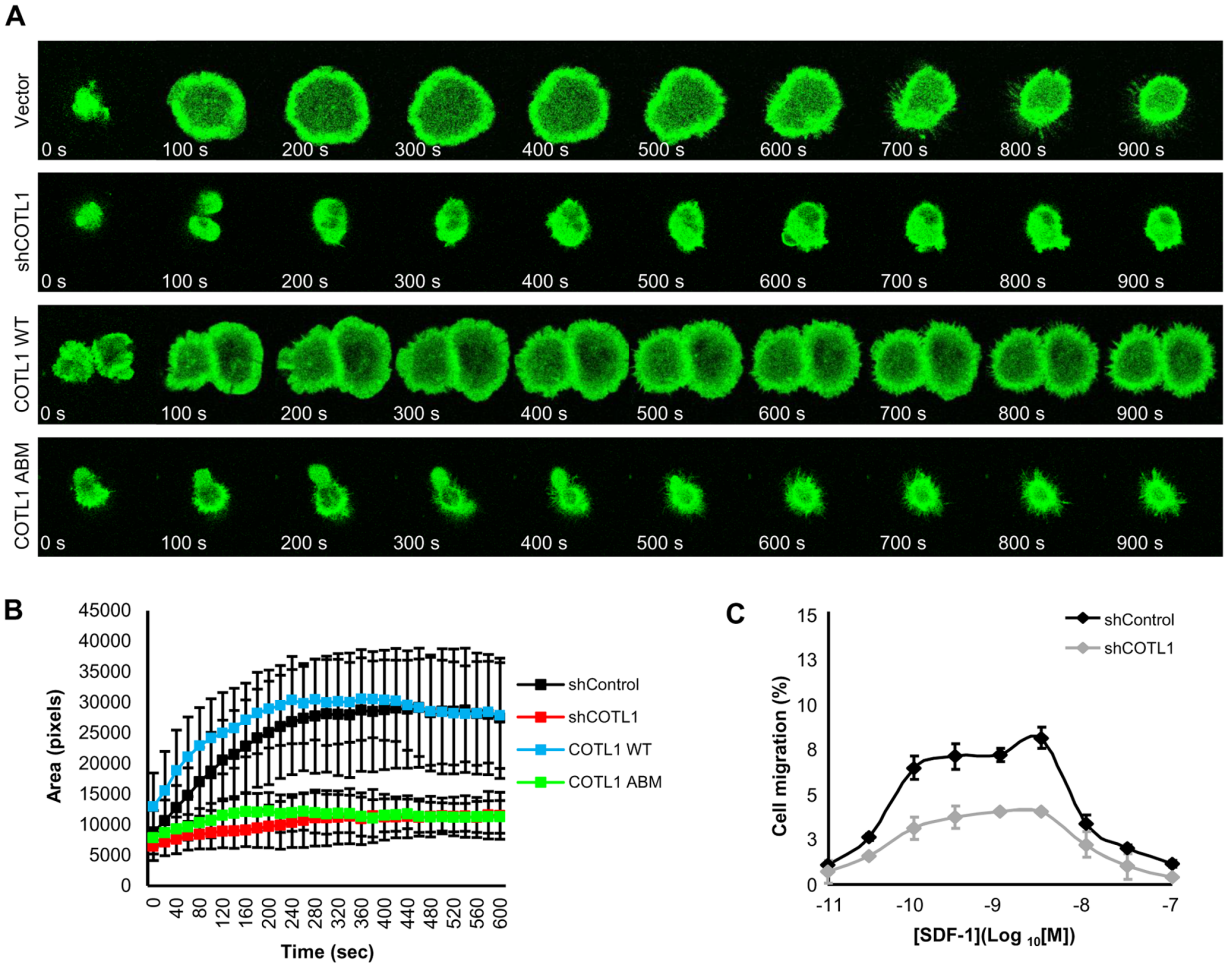
COTL1 regulates F-actin protrusion and T cell migration. (**A**) For F-actin spreading, Jurkat T cells stably expressing GFP-actin were transfected as in Figure **3E** and cherry positive cell images were captured by confocal microscopy following the initial contact with the anti-CD3 antibody-coated coverslip. Time-lapse image projections were acquired and single images representing 100 s time points following cell contact with the coverslip were shown. See associated Movies S5-S8. (**B**) The extent of anti-CD3 antibody-induced cell spreading (pixel area) was analyzed using ImageJ. 10 cells for empty vector, shCOTL1, COTL1 WT and COTL1 ABM were analyzed. Error bars represent SEM. (**C**) Dose-dependent migration in response to SDF-1 stimulation for control or COTL1-depleted Jurkat T cells. Each point denotes the mean percent of cells migrated ±SD; n  =  4. Results shown are representative of three independent experiments. COTL1 depletion was analyzed by immunoblot (unpublished data).

In order to further analyze the effect of COTL1 on lamellipodial protrusion, we directly visualized T cell spreading on coverslips in response to anti-CD3 stimulation and measured the spreading area [5]. GFP-actin Jurkat T cells were transfected with mCherry empty vector, shCOTL1 or suppression/re-expression plasmids as indicated in Figure 3 and dropped onto anti-CD3 antibody-coated coverslips. As expected, vector-transfected Jurkat T cells spread in a highly ordered fashion upon contact with the anti-CD3 antibody-coated coverslip forming a round lamellipodial structure that increased to nearly 3-times its starting area (Figure 4A and B). Spreading was typically maximal by 2 min, with retraction after about 5 min (Movie S5). DIC images showed membrane ruffling at the cell edge and radial retrograde flow within the lamellar region (Movie S5). In contrast, COTL1-depleted Jurkat T cells failed to spread upon contact, and instead, generated small filopodia-like spikes as well as membrane blebs devoid of F-actin that quickly retracted (Figure 4A, B and Movie S6). Significantly, the spreading defect was rescued by re-expression of COTL1 WT, but not COTL1 ABM (Figure 4A, B and Movies S7 and S8). In fact, expression of COTL1 ABM resulted in a phenotype similar to that seen upon depletion of COTL1. Taken together, these data indicate that COTL1 actin binding is required to sustain lamellipodial protrusion in response to TCR ligation.

The formation of lamellipodia is essential for cell migration [10], suggesting that COTL1 might regulate T cell migration via actin cytoskeleton rearrangement. To test this, we examined Jurkat T cell migration in response to stromal cell-derived factor-1 (SDF-1) stimulation [38]. As shown in Figure 4C, SDF-1-stimulated migration of control Jurkat T cells gradually increased up to 9% in a dose-dependent manner. In contrast, COTL1-depleted Jurkat T cells showed a decrease in migration in response to SDF-1 stimulation. Importantly, the levels of CXCR4 were unchanged between empty vector transfected- and COTL1-depleted Jurkat T cells (unpublished observations). Altogether, these data suggest that COTL1 is necessary for F-actin polymerization, contributing to efficient membrane protrusion and T cell migration.

### COTL1 antagonizes cofilin-mediated F-actin depolymerization

**Figure 5:**
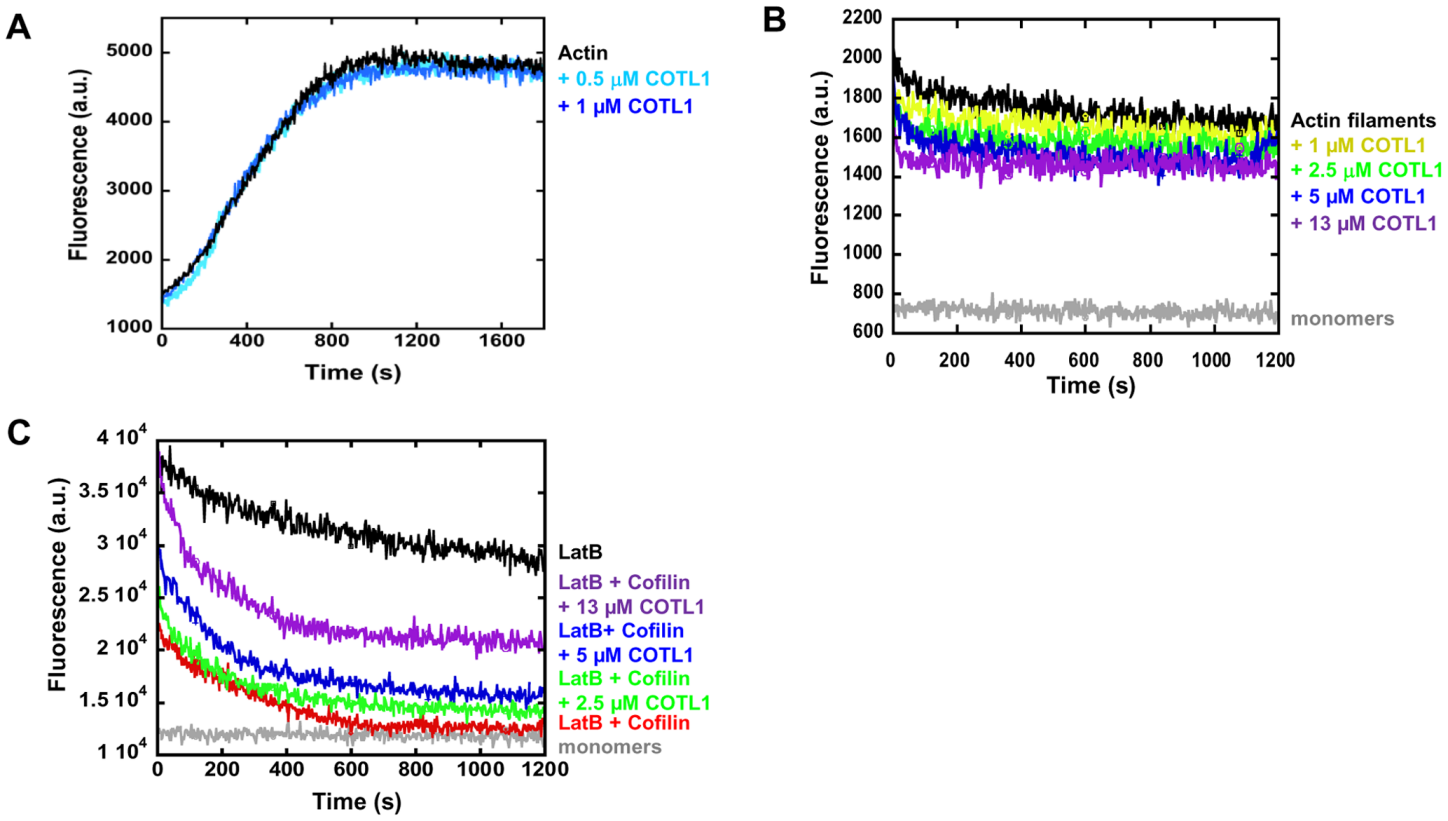
COTL1 antagonizes cofilin-mediated F-actin severing. (**A**) Pyrene-actin polymerization assay containing 4 µM actin monomers (5% pyrene-labeled actin) in the absence or presence of the indicated concentrations of COTL1. (**B, C**) Pyrene-F-actin depolymerization assay. 1.1 µM Actin (95% unlabeled and 5% pyrene-labeled actin) were polymerized for 2 h at room temperature and incubated with the indicated concentrations of COTL1 without (**B**) or with 0.5 µM cofilin and 20 nM Latruculin B (**C**). F-actin depolymerization was measured by fluorescent intensity of pyrene-labeled F-actin, n  =  3. Results shown are representative of three independent experiments performed in triplicate.

To delineate the mechanism by which COTL1 regulates actin dynamics, we examined whether COTL1 might promote actin polymerization or prevent F-actin depolymerization using pyrene-labeled actin. Consistent with previously published reports [39], [40], the addition of COTL1 did not accelerate F-actin polymerization *in vitro* (Figure 5A). Additionally, the addition of increasing concentrations of COTL1 did not lead to substantial depolymerization of F-actin that was seen by the addition of cofilin (Figure 5B and C). Since COTL1 appears to bind F-actin but does not promote either its polymerization or depolymerization, we hypothesized that COTL1 might protect F-actin from the severing and depolymerizing activities of cofilin. To examine this, we measured F-actin depolymerization in the presence of cofilin and increasing concentrations of COTL1. As can be seen in Figure 5C, the addition of Latrunculin B (LatB) to bind free actin monomers and prevent actin polymerization leads to slow depolymerization of F-actin over the 20 min timeframe of the experiment. In contrast, F-actin depolymerization could be substantially accelerated by the addition of cofilin, leading to near complete depolymerization by 10 min of incubation (Figure 5C, red line). Significantly, cofilin-mediated depolymerization could be attenuated by the addition of increasing concentrations of COTL1 (Figure 5C), suggesting that COTL1 can impair the depolymerizing and severing activities of cofilin.

### Depletion of both cofilin and COTL1 leads to lamellipodial protrusion at the IS

**Figure 6:**
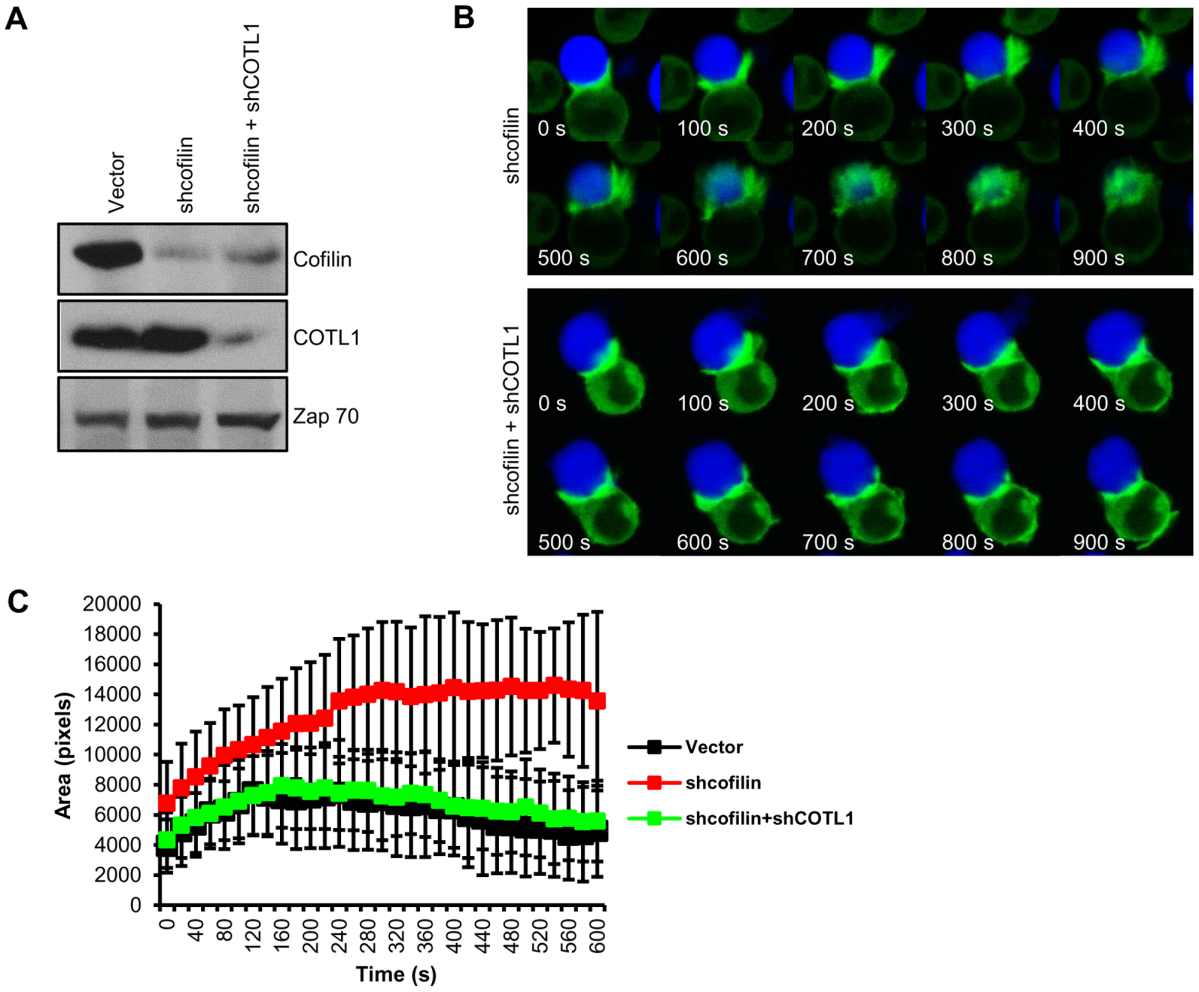
Depletion of cofilin generates excessive and longer lamellipodial protrusion at the IS, that is restored by co-depletion of COTL1. Jurkat T cells stably expressing GFP-actin were transfected with shcofilin alone or shCOTL1 and shcofilin. (**A**) Immunoblot showed suppression of cofilin or co-suppression of cofilin and COTL1. (**B**) GFP-actin-rich lamellipodial protrusion was monitored as described in Figure 3E. See associated Movies S9 and S10. (**C**) The lamellipodial protrusion (pixel area) at the cell-cell contact site was measured every 20 s for 10 min using Image J. Eight cells for each construct were analyzed. Error bars represent SEM.

Prior studies have indicated that the depletion of cofilin in other cell systems leads to exaggerated lamellipodia formation and F-actin accumulation as a result of diminished F-actin severing and depolymerization [16], [41], [42], [43]. In fact, we have found that TCR+CD28 stimulation leads to the association of both cofilin and COTL1 with the F-actin rich pellet (Figure 2D), and maybe in the absence of COTL1, F-actin polymerization and lamellipodial protrusion was blunted as a result of increased depolymerization and severing by cofilin. We therefore investigated the effect of cofilin depletion on lamellipodia formation at the IS. Consistent with prior studies in other cell types, the depletion of cofilin in Jurkat T cells resulted in the formation of a substantially increased and longer lamellipodial structure that surrounded the Raji B cell, which persisted for the entire length of imaging (Figure 6A and B upper and Movie S9). This is in contrast to empty vector-transfected cells, which showed lamellipodia formation that persisted up to 10 min followed by retraction and generation of an F-actin-rich band at the IS (Figure 3E top and Movie S1). Interestingly, consistent with the idea that COTL1 protects F-actin from the effect of cofilin, co-depletion of both cofilin and COTL1 resulted in the production of F-actin lamellipodial protrusions that were very similar to that seen in empty vector-transfected cells (Figure 3E top, 6B lower and Movies S1 and S10). The area encompassing the F-actin protrusion at the T cell-B cell contact site from eight individual movies for each transfectant was measured using Image J. As shown in Figure 6C, while the knockdown of cofilin substantially enhanced the area of F-actin and the duration of the response, the co-depletion of both cofilin and COTL1 produced an F-actin response similar to that of vector-transfected cells. Taken together, these data suggest that COTL1 and cofilin coordinate their activities to regulate the extent and duration of lamellipodial protrusion at the IS.

## Discussion

Herein we have shown that the ADF-H family member COTL1 localizes to the IS and interacts with F-actin following TCR+CD28 stimulation. In addition, we provide evidence that COTL1 is required for lamellipodia formation at the IS and in its absence only a band of polymerized F-actin accumulates. Significantly, we show that COTL1 can attenuate F-actin depolymerization mediated by cofilin, suggesting that the increased access of cofilin to F-actin in COTL1-depleted cells result in an inability to promote lamellipodial protrusion. Consistent with this notion, while depletion of cofilin results in excessive F-actin assembly and an extended duration of lamellipodial protrusion at the IS, depletion of both cofilin and COTL1 have a phenotype similar to that of empty vector-transfected cells. Taken together, our data suggest that these two members of the ADF-H family have opposing roles during F-actin generation at the IS, with COTL1 protecting newly generated F-actin from the severing and depolymerizing effects of cofilin, thereby promoting lamellipodia formation.

Both cofilin and COTL1 have an ADF-H domain, are structurally similar and can interact with F-actin [19]. However, it is well established that cofilin mediates lamellipodial extension and polarized cell migration by accelerating actin filament dynamics at the leading edge of migrating cells through its ability to both sever F-actin and promote filament depolymerization (13). In fact, depletion of cofilin in yeast, *Drosophila melanogaster* and mammalian cells results in excessive F-actin assembly and enhanced lamellipodial protrusion [16], [41], [42], [43], [44]. Consistent with these observations, we show that depletion of cofilin in Jurkat T cells results in excessive F-actin accumulation and a failure to retract the growing lamellipodia at the IS. In stark contrast, COTL1 depleted cells have an opposite phenotype showing an inability to develop lamellipodia and instead they only accumulate a thick band of F-actin at the cell–cell contact site. Prior biochemical studies have indicated that COTL1 does not sever F-actin or promote depolymerization like cofilin [20]. Our data are consistent with these reports. However, we provide new evidence that COTL1 and cofilin can compete for binding to F-actin and that COTL1 can abrogate cofilin-mediated F-actin depolymerization. Lastly, a recent study demonstrates a role for chicken coactosin in mediating F-actin polymerization, lamellipodia formation and directed cell migration of neural crest cells through its ability to promote F-actin polymerization [45]. These data are consistent with our findings in T cells, but we propose that in the absence of COTL1, there is excessive severing and depolymerization of F-actin by cofilin. This likely results in the generation of short branched F-actin filaments that are unable to support lamellipodia formation, but continue to accumulate at the IS due to the activation of proteins that promote F-actin assembly, such as WASP and WAVE2.

Cofilin is inactivated by LIMK1-mediated phosphorylation and activated by SSH1L-mediated dephosphorylation [19]. In untransformed human T cells, CD28 co-stimulation is required for dephosphorylation through a RAS-MEK and PI3K-mediated pathway that involves the serine/threonine phosphatase type 1 and 2 [46], [47], [48]. Thus, there are key mechanisms regulating the recruitment and the activation/inactivation of cofilin in T cells. Our data show that COTL1 is recruited to the IS and interacts with F-actin in a TCR+CD28-stimulated fashion. Whether COTL1 undergoes similar post-translational modification following T cell activation that effects its localization or F-actin binding activity will be of interest. In fact, COTL1 was first identified as an interacting partner of 5-lipoxygenase [49]. However, re-expression of COTL1mutant (K131A), known to impair the COTL1–5-lipoxygenase [50] interaction, rescued TCR-stimulated spreading and lamellipodia formation in COTL1-depleted cells (J.K. and D.D.B., unpublished observation). A recent study suggests that chicken coactosin functions downstream of activated Rac1 to promote lamellipodial protrusion and directed cell migration [45]. We have previously shown that the Arp2/3 regulator WAVE2, which is activated by Rac1, is required for T cell spreading and lamellipodia generation in response to TCR ligation [6]. Whether COTL1 is similarly recruited to the IS in a Rac1-dependent manner to facilitate WAVE2-dependent lamellipodia formation remains to be determined. Clearly, the identification of COTL1 interacting partners will provide further insight into the mechanisms by which this small F-actin binding protein is recruited to areas of polymerizing F-actin to sustain the growing lamellipodia.

In conclusion, we have shown that the ADF-H family member COTL1 was a critical regulator of F-actin dynamics at the IS, in part through its ability to bind F-actin and antagonize cofilin-mediated depolymerzation. While we were unable to identify a key signaling pathway that was defective in the absence of COTL1, previous studies using peptide inhibitors of cofilin F-actin binding have shown similar defects in TCR+CD28-stimulated expression of IL-2 and Interferon-γ [51]. Moreover, we have shown that actin dynamics were required for optimal IL-2 production in human T cells through TCR+CD28-stimulated expression of c-Rel and ultimately the binding to the CD28 RE/AP element within the IL-2 promoter [37]. Clearly, the use of COTL1 mutant animals will be an important step in uncovering the mechanism by which this protein can regulate not only F-actin dynamics in T cells, but also signaling pathways leading to IL-2 production.

## Supporting Information

Figure S1 COTL1 does not affect TCR-mediated inside-out signaling for integrin activation. **(A)** At 72 h post transfection with empty vector or shCOTL1, Jurkat T cells were co-stimulated with anti-CD3+CD28 antibodies for the indicated time points. Clarified cell lysates were prepared and protein was separated on the Tris-glycine SDS-PAGE gels and immunoblotted as indicated. **(B)** Jurkat T cells were transfected with GFP-tagged suppression plasmids and 72 h post transfection they were incubated for the indicated time at 37°C with CMAC-stained Raji B cells that were loaded (+) or unloaded (−) with SEE. Using two-color flow cyomtetry, the percentage of conjugated Jurkat T cells was calculated based on the simultaneous emission of both GFP and CMAC fluorescence. Presented data are an average of 3 independent experiments performed in triplicate. Error bars represent SEM.
(TIF)

Movie S1 Vector transfected GFP-actin Jurkat T cell interacting with SEE-pulsed Raji B cell.
(MOV)

Movie S2 shCOTL1 transfected GFP-actin Jurkat T cell interacting with SEE-pulsed Raji B cell.
(MOV)

Movie S3 shCOTL1 transfected GFP-actin Jurkat T cell re-expressing COTL1 WT interacting with SEE-pulsed Raji B cell.
(MOV)

Movie S4 shCOTL1 transfected GFP-actin Jurkat T cell re-expressing COTL1 ABM interacting with SEE-pulsed Raji B cell.
(MOV)

Movie S5 Vector transfected GFP-actin Jurkat T cell spreading in response to anti-CD3 stimulation.
(MOV)

Movie S6 shCOTL transfected GFP-actin Jurkat T cell spreading in response to anti-CD3 stimulation.
(MOV)

Movie S7 shCOTL1 transfected GFP-actin Jurkat T cell re-expressing COTL1 WT spreading in response to anti-CD3 stimulation.
(MOV)

Movie S8 shCOTL1 transfected GFP-actin Jurkat T cell re-expressing COTL1 ABM spreading in response to anti-CD3 stimulation.
(MOV)

Movie S9 shCofilin transfected GFP-actin Jurkat T cell interacting with SEE-pulsed Raji B cell.
(MOV)

Movie S10 shCOTL1 and shCofilin transfected GFP-actin Jurkat T cell interacting with SEE-pulsed Raji B cell.
(MOV)
