# Supplementary figures and images for: Coactosin-Like 1 Antagonizes Cofilin to Promote Lamellipodial Protrusion at the Immune Synapse
Source: PLoS One. 2014 Jan 13;9(1):e85090. doi: 10.1371/journal.pone.0085090 (PMC3890291; doi:10.1371/journal.pone.0085090)

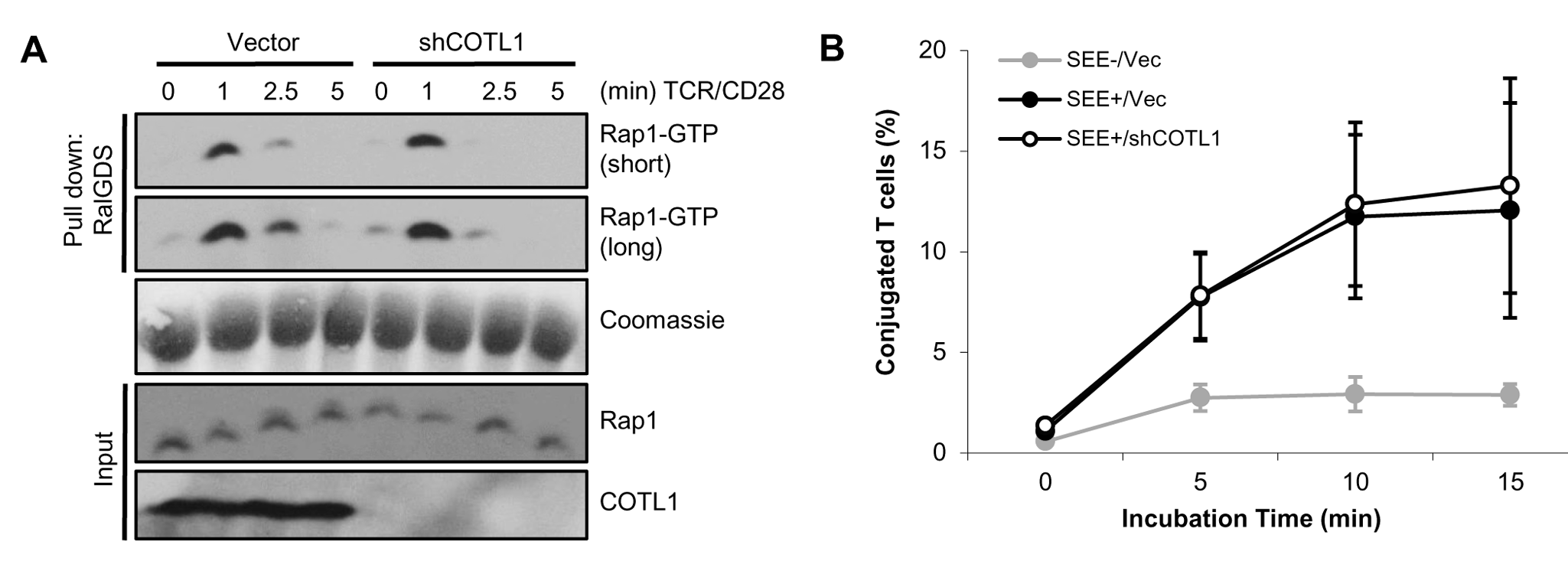

Supplement: Figure S1 — COTL1 does not affect TCR-mediated inside-out signaling for integrin activation. (A) At 72 h post transfection with empty vector or shCOTL1, Jurkat T cells were co-stimulated with anti-CD3+CD28 antibodies for the indicated time points. Clarified cell lysates were prepared and protein was separated on the Tris-glycine SDS-PAGE gels and immunoblotted as indicated. (B) Jurkat T cells were transfected with GFP-tagged suppression plasmids and 72 h post transfection they were incubated for the indicated time at 37°C with CMAC-stained Raji B cells that were loaded (+) or unloaded (−) with SEE. Using two-color flow cyomtetry, the percentage of conjugated Jurkat T cells was calculated based on the simultaneous emission of both GFP and CMAC fluorescence. Presented data are an average of 3 independent experiments performed in triplicate. Error bars represent SEM. (TIF) [file pone.0085090.s001.tif]
